# Prescriptions as quality indicators of pharmaceutical services in Polish community pharmacies

**DOI:** 10.1186/s12913-022-07772-2

**Published:** 2022-03-22

**Authors:** Marlena Ostrowska, Mariola Drozd, Rafał Patryn, Anna Zagaja

**Affiliations:** Department of Humanities and Social Medicine, Medical University of Lublin, ul. Chodźki 7, 20-093 Lublin, Poland

**Keywords:** Pharmacy, Pharmaceutical services, Law, Quality, Safety, Poland

## Abstract

**Background:**

Assessment of community pharmacies’ quality of service is a very difficult task, resulting from the multiplicity and variety of provided services as well as patient-related factors (i.e. their health condition, expectations, education level or cultural and social background). Although proceedings of pharmaceutical professionals are to a great extend legally determined and described in various acts and regulations, work diligence should be one of the most characteristic traits of a professional pharmacy employee. Many publications addressing the quality of services provided by pharmaceutical employees focus on patient satisfaction, here the authors focused on more objective methods i.e. prescription analysis.

**Objective:**

The main aim of the study was to assess whether post-inspection National Health Fund reports would constitute a reliable source of quality assessment of pharmaceutical services provided by community pharmacies.

**Methods:**

The study is an in-depth quality and quantity analysis of 28 post-inspection quarterly reports conducted by the National Health Fund between 2013 and 2019.

**Results:**

Vast majority of inspections ended in stating a variety of irregularities.

**Conclusions:**

The analysis of irregularities contained in the National Health Fund’s post-inspection reports does not seem an appropriate indicator of assessing the quality of pharmaceutical services provided in community pharmacies, because of its targeted character. Inappropriate performance of professional duties by staff members is the main source of irregularities in the implementation of prescriptions for reimbursable medications. There is a need to improve staffs’ professional competence and ultimately the quality of pharmaceutical services.

## Introduction

The definition of a pharmacy contained in the Polish Pharmaceutical Law Act determines that it is a healthcare entity, in which authorized persons (pharmacists and pharmaceutical technicians) provide pharmaceutical services [1]. Such services, according to art. 4.1(2) of the Act of December 2020 on the Profession of Pharmacist [2] are based on dispensing medicines and medical devices, as governed by the Pharmaceutical Law and the Act on Medical Devices [3].

Pharmacists in community pharmacies provide a number of services including supplying the society with medical products, counseling on prescriptions and self-treatment as well as providing pharmaceutical care, which in Poland is not yet a standard [4, 5]. Improving the quality of provided healthcare services is a part of daily work of professionals and quality indicators can prove useful for its assessment [6, 7]. Research on the quality of pharmaceutical care allowed for the development of numerous indicators e.g.. a Dutch study included 66 indicators within ten categories: ‘Quality management’, ‘Continuity of care’, ‘Communication with the patient’, ‘Clinical risk management’, ‘Compounding’, ‘Dispensing’, ‘Follow up of pharmacotherapy guidelines’, ‘Counseling’, ‘Logistics’, and ‘Training of pharmaceutical staff’ [8]. Developed and validated by the European Directorate for the Quality of Medicines and Health Care (EDQM) basic 4 sets of quality indicators cover 4 key areas of the pharmaceutical care process. These indicators can be used by regulatory authorities and healthcare professionals to evaluate pharmaceutical care practice and policy, and to promote effective and safe pharmacotherapy [9]. Evaluation of pharmaceutical services is key to ensuring and improving quality in pharmacists’ decision-making to optimize pharmacotherapy and its costs [10]. Available literature presents research results on the quality of pharmaceutical services in the sphere of pharmaceutical advice/ consultation and drug associated information [11–14]. One of the pharmaceutical services provided in pharmacies, which undergoes regular inspections and therefore can be assessed in terms of quality, is the fulfillment of prescriptions.The functioning of community pharmacies, due to their participation in the marketing of publicly funded products, is subjected to inspections of the National Health Fund (NFZ) – i.e. public payer responsible for e.g. providing product reimursement. Therefore [4]. inspections carried out by the NFZ determine inspection coverage and focus on i.e. correctness of the prescription in terms of formal requirements that are the basis for the reimbursement of medicines (e.g. data legibility, data completeness, correctly made and approved annotations),pricing of prescription medications, correctness of filling in prescriptions and confirmation of the right to fill them, providing data on the trade of reimbursable medications within established time limits and compliance with the regulations on the issue of reimbursed medications by professional personnel [15].

One of the main tasks of the Minister of Health in Poland is to provide the public with access to effective and safe medicinal products, and at the same time to reduce the costs of treatment for patients. Reimbursement is a tool for the implementation of this task, which also shapes the country’s drug policy. Pursuant to the Act on health care services financed from public funds, the right to reimbursed products (medications, foodstuffs for particular nutritional uses and medical devices) in Poland is granted to compulsorily insured persons, voluntarily insured persons and other persons (who meet the conditions specified in this act) [16] under the condition that an authorized person (doctor, dentist, medical assistant or senior medical assistant, nurse, midwife, and from April 2020, a pharmacist) issues the prescription.

A prescription for reimbursed drugs can be filled in any pharmacy (or pharmacy point) that has concluded an agreement with the National Health Fund. In order for the reimbursed drug to be issued with the correct payment, the prescription should be consistent with established requirements [7].

.The study aims to assess the quality of pharmaceutical services in community pharmacies through the prism of identified prescription associated irregularities found in the NFZ’s post-inspection reports.

### Material

The research material included documents – reports of inspections carried out in pharmacies by the provincial branches of the National Health Fund. The information is public and was published quarterly on the official website of the National Health Fund: [strona główna > O NFZ > kontrole” [17] [“home page > about the NFZ > inspections]. A total of 28 reports over a 7-year period (from 2013 to 2019) were analyzed in the study. Reports contained information on the number of inspections carried out in pharmacies concerning the implementation of reimbursable prescriptions. Each report contains information on the number of negatively assessed entities and proceedings in case of irregularities, and lists the types of identified irregularities in terms of prescription fulfillment. Analyzed NFZ reports did not specify the time scope of the inspection. Each date was chosen individually and related to the time period compliant with statutory provisions on the prescription’s storage time, i.e. 5 years from January 1 of the year following the year in which they were filled [1], usually not shorter than 1 year.

## Method

In order to evaluate the quality of pharmaceutical services provided by professional staff in publically accessible pharmacies, an in-depth quality analysis of the content of inspection reports carried out between 2013 and 2019, was conducted. Necessary documents were obtained in digital form from the official website of the Fund. The number of inspections is consistent with the number of inspected pharmacies. All data from the 28 quarters analyzed were obtained at the turn of 2019 and 2020. The first report used in the research was published on June 28, 2013 and it concerned the first quarter of 2013. The last one was published on March 9, 2020 and it encompassed the fourth quarter of 2019. The documents were collected, arranged, aggregated and their content was assessed in terms of filling prescription associated formalities. It was established that if an irregularity occurred in a given quarter, a value of 1 was attributed and if it did not that would account for 0 (−). This results from the factual structure of the analyzed inspection documents where the presence of the irregularity was stated, omitting information on the number of times the irregularity was detected in the audited quarter. Listed irregularities are grouped into 5 main categories, which concern and pertain to: formal data in the prescription, prescribed reimbursed medications, filling out of the prescription by an authorized person, the evaluation of the prescription and compliance of data submitted to the NFZ. Each of the categories encompasses subcategories which focus on individual irregularities found in the main category (Table 1).

**Table 1:** Categories and subcategories of stated irregularities

Irregularities category	Irregularities subcategories
Pertaining to formal data in the prescription	• lack, incomplete or illegible patient data on the prescription, • lack or incorrect data of the person authorized to issue the prescription and/or lack of a signature/stamp of that person, • lack or incorrect data of the issued prescription, no signature or stamp of the doctor on the introduced changes, • lack of payer ID or incorrect entry, • incomplete data in the „Healthcare provider” field, • lack of healthcare provider stamp and non-compliance of the print-over data on the prescription with the stamp of the person issuing the prescription.
Pertaining to prescribed reimbursed medications	• incorrect or incomplete data concerning prescribed medications, • prescribing psychotropic or narcotic drugs with other medications on one prescription, • incorrect prescribing of medications containing a psychotropic substance • and issuing a drug that was not on prescription.
Pertaining to filling out of the prescription by an authorized person	• incorrect fulfillment of prescriptions, i.e. not in accordance with the established pricing found in the applicable regulations, • providing medications with reimbursement despite its full payment status, • lack of confirmation of prescription fulfillment by the person issuing the medication, • incorrect confirmation of the patients’ additional entitlements, • double use of the same prescription, • double referral for reimbursement of the same prescription, • the implementation of prescriptions issued by unauthorized physicians.
Concerning the evaluation of the prescription and compliance of the data submitted to the National Health Fund	• inappropriate pricing of the medication, • lack of prescriptions based on, which the medication was issued, • failure to submit prescriptions for inspection, • providing inappropriate data from prescriptions to the National Health Fund, on the basis of which reimbursement is obtained, • delayed submission of reimbursement reports or corrections of these reports.

Table 1 Afterwards, a quantitative assessment of the identifies irregularities related to the number of performed inspections, was conducted. For statistical analysis, the Shapiro-Wilk test was used with the significance level set at α = 0.05, sample standard deviation.

## Results

**Fig. 1:**
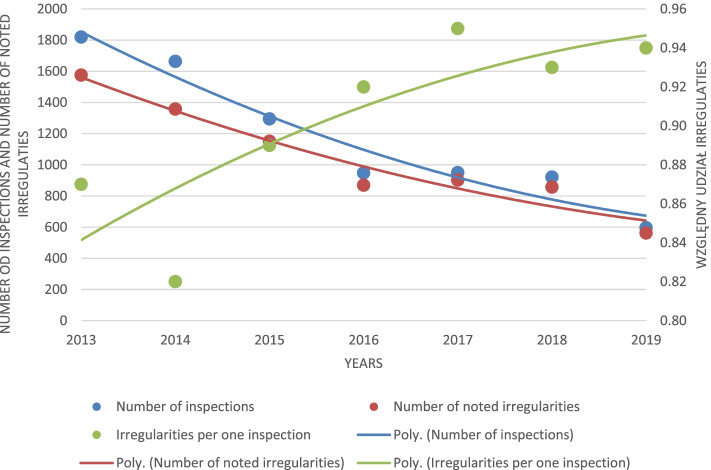
Trend in the number of inspections, detected irregularities and a relative input of irregularities between 2013 and 2019

The occurrence of irregularities in the inspected pharmacies and the number of inspections carried out between 2013 and 2019 are presented in Table 2. When analyzing those data, it is noticeable that the number of inspections carried out by the National Health Fund is decreasing, and there is also a downward trend in the number of detected irregularities. The coefficient of the relative share of the total number of identified irregularities to the number of performed inspections indicates an increase in the inspections’ effectiveness. According to the Shapiro-Wilk test, relative values of irregularities were normally distributed at the significance level of α = 0.05. The parameters of this distribution N (mi, sigma) N (0.90, 0.04) indicate a high mean number of defects and a low standard deviation. For relative values of irregularities, the confidence interval for the correlation coefficient was determined, which was also high (especially on the left-hand side) (0.175; 0.973), which results from the fact that the sample size is small - 7. Statistical analysis showed a very strong (almost complete) negative correlation (− 0.961) between the number of inspections in subsequent years (almost linear decrease in the number of controls) and a strong positive correlation (0.820) of detected irregularities and a strong negative correlation (− 0.869) between the number of controls and the share of irregularities, as shown in Fig. 1.

**Table 2:** Number of pharmacy inspections carried out by the National Health Fund between 2013 and 2019

	Number of pharmacies	Relative number of inspections per pharmacies	Number of inspections	Number of noted irregularities	Irregularities per one inspection
2013	12,221	0.149	1820	1576	0.87
2014	12,438	0.134	1664	1358	0.82
2015	12,740	0.102	1295	1151	0.89
2016	13,104	0.072	948	869	0.92
2017	13,338	0.071	950	902	0.95
2018	12,899	0.071	921	857	0.93
2019	12,286	0.049	596	562	0.94
R	–	−0.486	−0.869	−0.962	0.820
μ	–	0.093	1171	1039	0.903
SD	–	0.037	441.84	344.24	0.046

Based on data contained in Table 2, an average correlation can be noted between the number of inspections performed and the share of inspections in terms of the number of pharmacies (− 0,486). An assumption can be made that not every inspection was completed with post-inspection recommendations, which allows for the conclusion that there were pharmacies where no irregularities were spotted.

Information found in Table 3 is intended to show an incidence of detecting irregularities within individual categories occurring in a given year, and not the exact frequency of detecting specific irregularities in the course of the inspection procedures.

**Table 3:** Relative occurrence of selected categories of irregularities between 2013 and 2019 (‰)

Irregularities/ Year	2013	2014	2015	2016	2017	2018	2019	R	μ	SD
Pertaining to formal data in the prescription	7.69	13.22	18.53	25.32	16.84	17.37	26.85	0.7468	17.9747	6.6191
Pertaining to prescribed reimbursed medications	3.85	3.61	3.86	12.66	14.74	9.77	6.71	0.5365	7.8845	4.5732
Pertaining to filling out of the prescription by an authorized person	21.98	42.07	47.10	68.57	52.63	43.43	73.83	0.7258	49.9433	17.4113
Concerning the evaluation of the prescription and compliance of the data submitted to the National Health Fund	5.49	6.01	11.58	32.70	35.79	41.26	70.47	0.9508	29.0438	23.5026

Data contained in Table 3 allow for adopting a multidimensional perspective of carried out inspections. Statistical analysis revealed that in subsequent years relative values of all categories of irregularities are normally distributed according to the Shapiro-Wilk test, for α = 0.05. The distribution concerning the evaluation of the prescription and compliance of the data submitted to the National Health Fund shows an almost full correlation (linear correlation coefficient - 0.9508) between the number of performed inspections and identified irregularities, as well as the increase in the detection of these irregularities on an annual basis. This may be a confirmation that for the National Health Fund it is an important parameter, the most important due to the nature of the controlling body (NFZ), which guards public funds allocated in medication reimbursement. There are also no grounds to reject the hypothesis that this is a parameter on the basis of which the National Health Fund selects pharmacies for inspection.

It can be noticed that most irregularities arise at the stage of filling of the prescription and this parameter shows a very strong correlation (0.7258). A very strong correlation is also revealed concerning formal data in the prescription (0.7468) and an average on pertaining to prescribed reimbursed medications (0.5368). It should be concluded that the control system of the National Health Fund is improving, as there is a minimal probability of selecting a pharmacy for inspection that does not make any mistakes. The survey revealed irregularities in over 98.2% of the performed inspections with the simultaneous decrease of performed inspections.

## Discussion

The study revealed a systematic decrease (since 2013) in the number of pharmacy inspections as well as in the number of identified irregularities. However, attention should be paid to the ratio of identified irregularities to the number of inspections performed. This is evidenced by the coefficient in Table 2, which is the quotient of the total number of identified irregularities by the number of inspections. The higher the value of the coefficient, the greater the average number of detected irregularities per individual inspection. Based on obtained data, it can be noticed that not every inspection was completed with post-inspection recommendations. This allows us to conclude that there were also inspections, which revealed no irregularities, which in turn allows us to draw a conclusion that there were pharmacies that worked properly within the inspected areas. Obtained data reveal the multidimensionality of inspections carried out by the National Health Fund in pharmacies.

During the analysis, irregularities were found on many levels. Most of the irregularities arise at the stage of fulfilling the prescription i.e. by an authorized person (pharmacist or a pharmacy technician). Failure to comply with the rules of carrying out professional activities results in the low level of expertise of the staff in the implementation of prescriptions. Expertise in this sense concerns meticulousness and commitment to performing professional activities.

Holdford D. and Schulz R. investigated the impact of technical quality of pharmaceutical services on patient’s perception. The results indicated that positive patient feedback is most affected by functional quality i.e., whether patients y receive the drug or not. This is also the case when the quality of service is poor or the pharmacist makes an error. Therefore, researchers concluded that reliance on patient feedback in determining the quality of pharmaceutical services has its limitations. Patients opinion are essential, however that cannot be the sole premise for a professional assessment of the quality of provided pharmaceutical services [18]. Our study leads to similar conclusions, however, on the basis of post-inspection reports and not patient’s opinions. What needs to be underlined is that although the patient may be satisfied with the provided pharmaceutical services, their poor quality in the area of prescription fulfillment may in fact have an impact on that patient’s health and finances. The existence of irregularities in terms of pricing of the prescriptions medicines and the compliance with data transferred to the NFZ in the form of reports increases yearly. That is why professionalism of people filling prescriptions is of great importance.

On the other hand, irregularities on the side of the person issuing the prescription (physician, dentist, nurse, midwife [19]), indicates a low level of professionalism of that professional. These irregularities include irregularities in terms of formal data necessary to issue a prescription for reimbursable drugs i.e. patient’s data data of the person issuing the prescription or data of the healthcare provider. These irregularities are a result of non-compliance with legal provisions, which precisely define such matters [1]. Irregularities in terms of data pertaining to the person issuing the prescription such as failure to comply with the obligation to sing and stamp corrections made on the prescription and the lack of identifier of an appropriate NFZ department are further violations of the required rules in this regard. In this area, most frequent are incorrect data concerning the patient and the person authorized to issue the prescription, as well as missing or incorrect date of issue of the prescription. Because some of these irregularities can be corrected by the pharmacy staff, this category of non-compliance is attributed to pharmacists and pharmaceutical technicians. In this case, it is worth to underline that signatures on the documentation are strictly required, so that the activity expressed and described therein (confirmation of sale and delivery of drugs to the authorized entity) is valid.

What is more, data on the frequency of irregularities in terms of prescribed reimbursed drugs were analyzed. Irregularities in this respect are also the result of non-compliance with legal provisions by the issuer of the prescription. Any deficiencies in the scope of prescribing psychotropic drugs are in breach of the provisions of the Regulation of the Minister of Health, which strictly specifies the conditions for issuing prescriptions containing narcotic drugs and psychotropic substances [20]. The most common misconduct in this category is incompleteness or missing data related to the prescribed medication. The violation of regulations in this respect was detected in as many as 27 of the 28 reports from the period covered by the study. Eight reports stated that the psychotropic drug was prescribed on the same prescription with another drug, which is also inconsistent with applicable regulations. Incorrect prescription of drugs containing a psychotropic substance was identified in 9 reports. Such events may be related to professional practice including prescribing medication error, that may cause or lead to inappropriate medication use and result in patient harm [21]. In each analyzed period, irregularities in the scope of filling prescriptions were detected. Those prescriptions did not meet formal requirements associated with necessary data i.e. patient’s and those issuing the prescription. Also in each analyzed report, during the course of the inspection, the fulfillment of the prescription was finalized despite incorrect or missing date of issue. Errors associated with inaccurate or incomplete prescription data and pricing prescription medicines have occurred in each of the reports. Identified errors made by pharmacy staff were mechanical. A mechanical error is a mistake in dispensing or preparing a prescription, such as administering an incorrect drug or dose, or dispensing the incorrect dose, quantity, or strength [21].

Also, irregularities were identified related to the lack of supervision by the pharmacy manager over the pharmacy staff. The most frequent irregularity was fulfilling prescriptions by unauthorized employees (e.g. pharmaceutical technicians dispensing psychotropic drugs). Conducted studies revealed that the irregularities largely relate to the level of professionalism of those working in the pharmacy, which may depend, among others, on the increased amount of workload, and lack of staff.

The subjects’ scope was the object of previous research, which should be mentioned in the discussion. The research of Lithuanian scientists illustrated the analysis of the attitudes of professional pharmacy personnel in relation to the quality of pharmaceutical services. Four hundred seventy-one specialists took part in the study. Two main sectors have been distinguished in which the quality of services was assessed counseling in the field of pharmacotherapy (providing information about the drug, side effects, health promotion) and socio-economic aspects (action taking into account the needs and financial possibilities of the patient). Respondents assessed both of the aspects positively, however they stated that the pharmacotherapeutic aspect (advisory) were at a lower level. Results varied depending on gender, age, qualifications (Master of Pharmacy / Pharmaceutical Technician) size and the pharmacies location, which may suggest that patients’ needs may differ depending on their place of residence [22].

The Lithuanian conclusions are confirmed by Polish results indicating a relatively low quality level of pharmacotherapeutic counseling, regardless prescription drug dispensing [11] or self-treatment counseling [12–15]. This also allows to assume that pharmacists have different abilities and skills to provide pharmaceutical services depending on the location of the pharmacy in which they work [22]. One of the pharmaceutical services provided in pharmacies in many European countries is pharmaceutical care. Research indicates that the level of Polish patient’s satisfaction with provided pharmaceutical services is lower, which may result from the fact that pharmaceutical care is practically not functioning in the country. Higher quality of the provided services e.g. in the form of greater interest of the pharmacist in the patient or professionalism of service, could contribute to increased patient satisfaction [23]. The concept of lack of professionalism of the pharmacy staff and their work to a large extend concerns the failure to respect legal provisions, which are equipped with appropriate content describing the professional conduct of a pharmacist and pharmaceutical technician as in the case of issuing reimbursed or psychotropic drugs.

Analysis of the quality of pharmaceutical services provided in community pharmacies may be performed on the basis of patient surveys or by surveying pharmacy professionals in this area through, among others participant observation research i.e. “secret shopper”. However, such studies are largely based on subjective feelings of patients or customers. The importance of the service for the patient can often only refer to the availability of the drug and for a pharmacy employee, among others, transaction efficiency. Reports and compilations of data from official documents made by entities authorized to conduct inspections are free from the element of “subjectivity”. Therefore, the analysis of impartial documents resulting from the inspection provides knowledge about occurring irregularities and are not judgmental or opinion based. Data collected over time can be interpreted directly, as a quantitative reflection of a specific process that took place. In this case, this reflection pertained to categorized irregularities in terms of the provision of pharmaceutical services in community pharmacies. Jakobs S et all believe that they have important implications for community pharmacies, highlighting the importance of organisational culture, staffing and skill mix for maximising the delivery of high-quality pharmacy services [24].

Current research revealed that the medical personnel responsible for patients’ pharmacotherapy and often their inadequate quality of work is the source of many irregularities detected in Polish community pharmacies. Numerous irregularities on many levels were noted in terms of filling out prescriptions, both on the side of the person writing and in consequence, the person fulfilling the prescription. Many of those irregularities concerned non-compliance / ignorance of legal provisions by both doctors and pharmacists or pharmaceutical technicians. According to Ibrahim O.M. et. all there is a need to improve the education of community pharmacists and physicians to ensure safe dispensing practice and introduce of solutions to decrease the number of errors and hence reduce patient’s heath risk [25]. It is estimated that the number of stated irregularities in the fulfillment of prescriptions, should decrease in the upcoming years. This should be closely related to the introduction of electronic prescriptions from January 8, 2020.

## Conclusion

The analysis of identified irregularities contained in the National Health Fund’s post-inspection reports carried out in community pharmacies does not seem an appropriate indicator for assessing the quality of pharmaceutical services provided by professional personnel. Because of its targeted character, only pharmacies that make the most errors will be inspected more frequently, which only to some extent serves quality improvement. Inappropriate performance of professional duties by staff members is the main source of irregularities in the implementation of prescriptions for reimbursable medications. The study’s results allow for the assessment of errors related to the fulfillment of prescriptions. This in turns allows for defining most commonly made errors and could be used in training the pharmacy staff in the do’s and dont’s of drug prescriptions, leading to the improvement of provided services and patient safety. Such trainings should be conducted on a regular basis.

## Data Availability

The datasets generated and/or analysed during the current study are available in the FNZ repository [https://www.nfz.gov.pl/o-nfz/kontrole/].
